# Microbiome-targeted antibiotics provide no additional microbiologic or inflammatory benefit during cystic fibrosis pulmonary exacerbations: results from the CFMATTERS trial

**DOI:** 10.1093/ajrccm/aamag336

**Published:** 2026-06-29

**Authors:** Gisli G Einarsson, Andrew J Lee, Zina Alfahl, Lauren Kerrigan, Joseph A Eustace, Sinéad Weldon, Barry J Plant, J Stuart Elborn, Clifford C Taggart, Marcus A Mall, Michael M Tunney

**Affiliations:** School of Pharmacy, Queen’s University Belfast, Belfast, United Kingdom; School of Pharmacy, Queen’s University Belfast, Belfast, United Kingdom; School of Pharmacy, Queen’s University Belfast, Belfast, United Kingdom; Airway Innate Immunity Research (AiiR) Group, Wellcome-Wolfson Institute for Experimental Medicine, Queen’s University Belfast, Belfast, United Kingdom; Cork Centre for Cystic Fibrosis (3CF), Cork University Hospital, University College Cork, Cork, Ireland; The Health Research Board-funded Clinical Research Facility, University College Cork, Cork, Ireland; Airway Innate Immunity Research (AiiR) Group, Wellcome-Wolfson Institute for Experimental Medicine, Queen’s University Belfast, Belfast, United Kingdom; Cork Centre for Cystic Fibrosis (3CF), Cork University Hospital, University College Cork, Cork, Ireland; The Health Research Board-funded Clinical Research Facility, University College Cork, Cork, Ireland; Department of Medicine, University College Cork, Cork, Ireland; School of Medicine, Dentistry and Biomedical Sciences, Queen’s University Belfast, Belfast, United Kingdom; Airway Innate Immunity Research (AiiR) Group, Wellcome-Wolfson Institute for Experimental Medicine, Queen’s University Belfast, Belfast, United Kingdom; Department of Pediatric Respiratory Medicine, Immunology and Critical Care Medicine, Charité-Universitätsmedizin Berlin, Berlin, Germany; German Center for Lung Research (DZL), associated partner site Berlin, Berlin, Germany; German Center for Child and Adolescent Health (DZKJ), partner site Berlin, Berlin, Germany; Cluster of Excellence ImmunoPreCept, Charité-Universitätsmedizin Berlin, Berlin, Germany; School of Pharmacy, Queen’s University Belfast, Belfast, United Kingdom

*To the Editor*

In people with cystic fibrosis (CF), acute pulmonary exacerbations (PEx) are typically treated with a 14-day regimen of dual intravenous (IV) antibiotics, such as ceftazidime and tobramycin.^1^^,^^2^ Advances in culture-independent sequencing have revealed complex polymicrobial airway communities in CF,^3^^,^^4^ prompting the hypothesis that targeting additional taxa identified by next-generation sequencing (NGS) may enhance treatment efficacy. The Cystic Fibrosis Microbiome-directed Antibiotic Therapy Trial in Exacerbations Results Stratified (CFMATTERS) evaluated an NGS-based approach for the treatment of PEx in adults with CF chronically infected with *Pseudomonas aeruginosa*. The addition of a third antibiotic, selected on the basis of historic sputum microbiome analysis, did not improve clinical outcomes compared with usual dual antibiotic therapy.^5^ However, it is unclear whether this approach results in a meaningful additional reduction in microbiologic measures or inflammatory markers during PEx. The aim of this study was to determine whether adding a microbiome-directed antibiotic to standard therapy alters the sputum microbiome and airway inflammation during PEx in CF.

## Methods

Adults with CF chronically infected with *P. aeruginosa* who experienced an eligible PEx and had sputum collected at baseline (stable state), day 0 (initiation of IV therapy), and day 14 (completion of therapy) were included. Participants received usual therapy (guideline-recommended dual IV therapy of ceftazidime or aztreonam plus tobramycin)^2^ or the same regimen plus an additional antibiotic selected by expert consensus targeting genera identified by prior NGS profiling.^2^^,^^5^

Participants were included if they had matched microbiome samples at baseline, day 0, and day 14 and received antibiotics per protocol; excluded participants had similar baseline clinical characteristics. Sputum microbiota were characterized by 16S rRNA marker-gene sequencing on the Illumina MiSeq platform^3^^,^^6^ with total bacterial density (16S rRNA copies/mL) quantified by qPCR. Relative abundance and alpha-diversity were derived from sequencing data. Human neutrophil elastase (HNE), a marker of neutrophilic inflammation, was measured when sample volume permitted; these analyses are exploratory due to incomplete sample pairing across visits. Longitudinal changes were analyzed using mixed-effects models with a subject-specific random intercept and fixed effects for visit, treatment, and their interaction, accounting for within-subject changes over time.

## Results

One hundred one participants (51 usual therapy; 50 microbiome-directed therapy) with matched microbiome samples at baseline, day 0, and day 14 were analyzed, representing 68% of the CFMATTERS PEx cohort (Table 1).

**Table 1:** Baseline demographic characteristics, microbiome metrics, and inflammatory biomarker profiles of participants (*N* = 101) in the exacerbation cohort.

Characteristic	Usual therapy (*n* = 51)	Microbiome-directed (*n* = 50)	Total (*N* = 101)	*P* value
**Age, y, median (IQR)**	30.1 (26.6-37.9)	28.3 (25.7-33.5)	29.3 (26.0-36.1)	.195
**Sex, No. (%)**				
** Male**	28 (54.9)	17 (34.0)	45 (44.6)	
** Female**	23 (45.1)	33 (66.0)	56 (55.4)	.056
**FEV_1_, % predicted, median (range)**	44.5 (23.4-86.2)	45.4 (26.3-93.1)	45 (23.4-93.1)	.757
**Total bacterial density, log_10_ 16S rRNA copies/mL, median (range)**	7.4 (6.1-8.7)	7.5 (6.4-8.3)	7.5 (6.1-8.7)	.812
**Shannon–Wiener Index (H), median (range)**	0.6 (0-2.4)	0.2 (0-1.7)	0.4 (0-2.4)	.031
***Pseudomonas* spp. relative abundance, %, median (range)**	80.6 (0.1-100)	96 (0.1-99.8)	89.1 (0.1-100)	.014
**HNE, ng/mL, median (range)^a^**	195 (0 -10 154)	834 (0 -11 480)	543 (0-11 480)	.225
**Ivacaftor, No. (%)**				1.000
** Yes**	4 (7.8)	3 (6.0)	7 (6.9)	
** No**	47 (92.2)	47 (94.0)	94 (93.1)	
**Days from baseline to day 0, median (IQR)**	80 (61.5-122.5)	91 (63-122)	NA	.689
**Antibiotics at day 0, No. (%)**				
** Tobramycin-ceftazidime**	38 (74.5)	36 (72)	74 (73.3)	
** Tobramycin-aztreonam**	13 (25.5)	14 (28)	27 (26.7)	
**Third antibiotic recommended and administered, No. (%)^b^**				
** Yes**	NA	48 (96)	48	
** Different**	NA	2 (4)	2	

Abbreviations: HNE, human neutrophil elastase; IQR, interquartile range; NA, not applicable.

a For HNE, usual therapy had 16 missing measurements and microbiome-directed therapy had 25 missing measurements.

b For microbiome-directed therapy, on 2 occasions a different antibiotic was administered from what was recommended, ie, doxycycline and amoxicillin once each. Third antibiotic administered: moxifloxacin = 18; co-amoxiclav = 13; doxycycline = 11; meropenem = 3; co-trimoxazole = 2; amoxicillin = 1; fosfomycin = 1; vancomycin = 1.

To assess whether baseline microbiome profiles remained ­representative at treatment initiation, paired baseline and day 0 samples were examined using within-subject Bray–Curtis dissimilarity and dominant genus concordance. Dissimilarity was low to moderate in both the microbiome (median, 0.090 [IQR, 0.016-0.329]) and usual therapy (median, 0.187 [IQR, 0.049-0.491]) arms, with a nonsignificant trend toward lower dissimilarity in the microbiome-directed arm (Wilcoxon rank-sum test, *P* = .058). At the taxonomic level, the dominant genus was preserved in 82 of 101 (81.2%) sample pairs. Where it changed, the original dominant taxon was still detectable in 18 of 19 samples at lower relative abundance, suggesting shifts in dominance rather than complete community replacement. Together, these findings suggest that baseline microbiome profiles were broadly representative at treatment initiation.

### Microbial response to IV therapy

IV antibiotic treatment was associated with significant but transient perturbation of the sputum microbiome in both treatment arms.^7^^,^^8^ Total bacterial density decreased during treatment (*P* = .014 for visit effect) (Figure 1A), with similar absolute reductions observed across groups. *Pseudomonas* spp. relative abundance changed significantly over time (*P* <.001), decreasing at day 14 in both groups (Figure 1B). However, at follow-up (day 28 and month 3), microbiota patterns reverted toward preexacerbation profiles (data not shown).^9^

**Figure 1:**
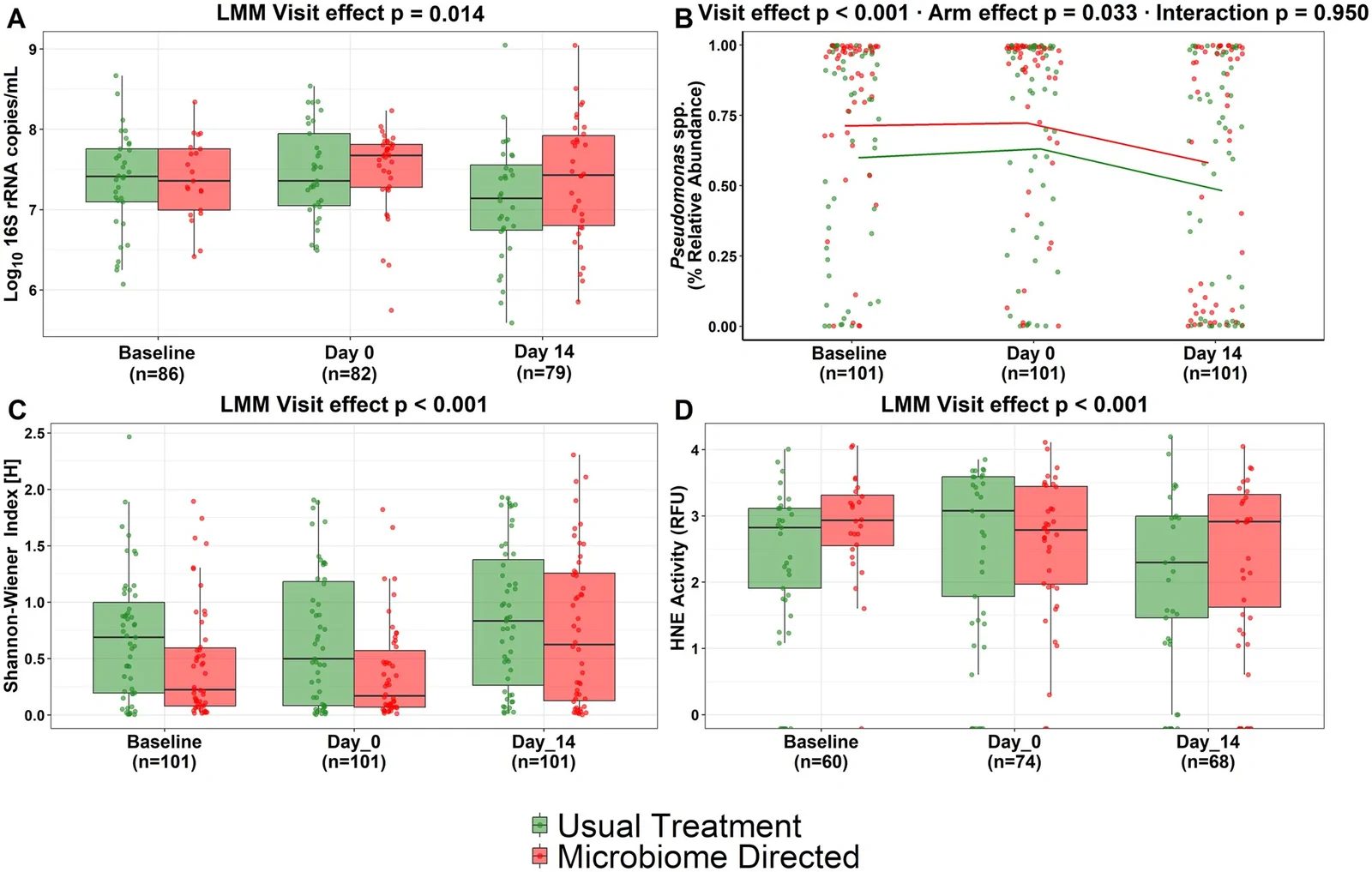
Longitudinal microbiological and inflammatory changes during treatment of cystic fibrosis pulmonary exacerbation. (A) Airway bacterial load expressed as log_10_ 16S rRNA gene copies/mL at stable baseline, day 0 (start of exacerbation treatment), and day 14 (end of treatment). (B) Relative abundance of *Pseudomonas* spp. (%) across visits. Lines represent group-level means over time. (C) Alpha diversity measured by Shannon–Wiener index (H). (D) HNE activity (RFU). Participants received either usual antimicrobial therapy (green) or microbiome-directed therapy (red). Boxplots represent median and interquartile range (IQR), with whiskers extending to 1.5 × IQR; individual observations are shown as jittered points. *P* values displayed above panels reflect fixed effects from linear mixed-effects models accounting for repeated measures within participants. Panel (B) additionally displays treatment arm and treatment × visit interaction effects. Sample sizes for each visit are shown on the x-axis. Abbreviations: HNE, human neutrophil elastase; IQR, interquartile range; LMM, linear mixed model; RFU, relative fluorescence units.

Despite baseline differences, no visit × arm interaction was observed, and model-based estimates showed no differences between arms at any visit (all *P* >.05), indicating baseline imbalance rather than differential treatment response.

Shannon diversity changed significantly over time (*P* <.001), consistent with transient treatment-related shifts, but no visit × treatment interaction was observed (*P* = .420), indicating similar longitudinal trajectories across arms (Figure 1C). Adding a microbiome-directed third antibiotic did not further reduce bacterial burden or produce sustained shifts in community structure versus standard dual therapy (Figure 1A-C).

### Inflammatory response

HNE activity declined by day 14 (*P* <.001; Figure 1D), consistent with reduced neutrophilic inflammation following IV therapy. There was no treatment or visit × arm effect, indicating similar trajectories across groups despite higher overall HNE activity in the microbiome-directed arm.

## Discussion

To our knowledge, this represents the first randomized evaluation of microbiome-directed antibiotic therapy in CF exacerbations. Standard dual IV therapy induced measurable but reversible changes in sputum microbial ecology and airway inflammation. Reductions in total bacterial burden and relative abundance of *P. aeruginosa*, despite its continued predominance, were accompanied by transient increases in diversity and decreases in inflammatory markers, consistent with effective antimicrobial treatment.^7^^,^^8^ Importantly, adding a third antibiotic, targeting the second, third, and fourth most prevalent bacterial genera, did not provide additional benefit in altering microbial community structure, relative abundance, or inflammation compared to usual therapy. These findings align with the clinical outcomes of the CFMATTERS trial, which reported no difference in lung function, time-to-next PEx, and symptom burden between treatment groups.^5^

The limited impact of the third antibiotic on the sputum microbiome may reflect the underlying characteristics of the study population and the antibiotic regimens used. Participants had low-diversity, *P. aeruginosa*–dominated microbiomes, which are typically less responsive to antibiotics, suggesting that the lack of additional microbiome modulation was not due to the third antibiotic itself. Moreover, both treatment groups received broad-spectrum antibiotics, primarily ceftazidime and tobramycin, with activity against a wide range of microbial taxa. Therefore, any potential impact of the third, NGS-guided antibiotic on less abundant microbial genera may have been obscured.^10^

These findings highlight ecological constraints inherent in advanced CF lung disease. Once airway communities become dominated by established pathogens, further antibiotic intensification may have limited capacity to reshape the microbial ecosystem over short treatment intervals. This apparent biological ceiling effect provides mechanistic context for the neutral clinical outcomes in CFMATTERS, including no improvement in lung function, time to next exacerbation, or symptom burden from adding a third microbiome-targeted antibiotic in people with CF.^5^

In summary, microbiome-directed therapy for PEx did not significantly alter airway microbial community structure or inflammation compared to usual therapy. These findings do not support routine addition of antibiotics based solely on sputum NGS profiling in chronically *P. aeruginosa–*colonized adults. Future approaches may require earlier intervention, alternative targets, or host-directed strategies rather than further antibiotic intensification.

## Supplementary Material

aamag336_Supplementary_Data
